# Prevalence, Mass, and Glucose-Uptake Activity of ^18^F-FDG-Detected Brown Adipose Tissue in Humans Living in a Temperate Zone of Italy

**DOI:** 10.1371/journal.pone.0063391

**Published:** 2013-05-08

**Authors:** Agnese Persichetti, Rosa Sciuto, Sandra Rea, Sabrina Basciani, Carla Lubrano, Stefania Mariani, Salvatore Ulisse, Italo Nofroni, Carlo Ludovico Maini, Lucio Gnessi

**Affiliations:** 1 Section of Medical Pathophysiology, Food Science and Endocrinology, Department of Experimental Medicine, University of Rome “La Sapienza”, Rome, Italy; 2 Department of Nuclear Medicine, Regina Elena National Cancer Institute, Rome, Italy; 3 Department of Public Health and Infectious Diseases, Medical Statistics and Biometry, University of Rome “La Sapienza”, Rome, Italy; Azienda Policlinico S. Orsola-Malpighi, Italy

## Abstract

**Background:**

The ^18^F-fluorodeoxyglucose (^18^F-FDG)-detected brown adipose tissue (BAT), is enhanced by cold stimulus and modulated by other factors that still have to be disentangled. We investigated the prevalence, mass, and glucose-uptake activity of ^18^F-FDG-detected BAT in a population of adults living in the temperate climatic zone of the Rome area.

**Methods and Findings:**

We retrospectively analyzed 6454 patients who underwent^ 18^F-FDG positron emission tomography/computed tomography (PET/CT) examinations. We found ^18^F-FDG BAT in 217 of the 6454 patients (3.36%). Some of them underwent more than one scan and the positive scans were 278 among 8004 (3.47%). The prevalence of patients with at least one positive scan was lower in men (1.77%; 56 of 3161) compared with women (4.88%; 161 of 3293). The BAT positive patients were most frequently younger, thinner and with lower plasma glucose levels compared with BAT negative patients. The amount of BAT in the defined region of interest, the activity of BAT and the number of positive sites of active BAT were similar in both sexes. The prevalence of patients with ^18^F-FDG positive PET/CT was highest in December-February, lower in March-May and September-November, and lowest in June-August and was positively correlated with night length and negatively correlated with ambient temperature. Changes in day length and variations of temperature, associated with the prevalence of positive BAT patients. Among the patients who had multiple scans, outdoor temperature was significantly lower and day length was shorter on the occasion when BAT was detected.

**Conclusions:**

This study identifies day length, outdoor temperature, age, sex, BMI, and plasma glucose levels as major determinants of the prevalence, mass, and activity of ^18^F-FDG-detected BAT.

## Introduction

Brown adipose tissue (BAT) is a an energy-expending organ that generates heat [1]. BAT helps maintaining core temperature in a cold environment without shivering. Its thermogenic potential is due to the uncoupling protein 1 (UCP1), a transmembrane protein of the inner mitochondrial membrane of the brown fat cells. BAT thermogenic activity and involvement in energy balance depend on the adrenergic stimulation of brown adipocytes [2]. Recent studies, using non invasive ^18^F-fluorodeoxyglucose (^18^F-FDG) positron emission tomography (PET)/computed tomography (CT) scanning, demonstrated that, in humans, BAT is expressed in adults, besides newborn infants [3]–[8].

In adults, the prevalence of ^18^F-FDG positive BAT is variable, ranging between 1.72% and 6.85% in studies involving cancer patients [4], [9]–[16], whereas in cold-exposed subjects, the prevalence was reported to be close to 100% [5], [6]. Ambient temperature at the time of the PET/CT assessment is a critical factor to detect BAT [4]–[6], [8]–[10], [14], [16]. Furthermore, factors such as sex [4], [9], [10], [13], [14], [17], [18], age [4], [7]–[9], [13], [14], [17], [18], body mass index (BMI) [5], [7], [8], [18], plasma glucose [4], and day length [10] are all important determinants of ^18^F-FDG uptake in BAT although their relative importance is still controversial.

Here, we examined prevalence, mass, and glucose-uptake activity of ^18^F-FDG-detected BAT in a large cohort of subjects who underwent PET/CT examination for cancer diagnosis and staging in the temperate climate of Rome.

## Methods

### Ethics Statement

The project was approved by the Institutional Review Board of the Dipartimento di Prevenzione e Diagnostica Oncologica, UOC di Medicina Nucleare, Istituto Nazionale Tumori Regina Elena, Roma. The data were analyzed anonymously. Written consent was given by the patients or from the next of kin, caretakers, or guardians on the behalf of the minors/children participants for their information to be stored in the hospital database and used for research.

### Subjects and Methods

A total of 6454 patients (3293 women, 3161 men; age range 2–94 yr) underwent 8004 consecutive ^18^F-FDG PET/CT scans (4087 women, 3917 men) from January 2007 to June 2010 at the Istituto Nazionale dei Tumori Regina Elena (Rome, Italy; 41.81°N 12.45°W). The large majority of scans were for cancer detection. All PET/CT scans were performed using a Biograph 16 High Rez PET/CT scanner (SIEMENS AG, Munich, Germany). Patients came from the metropolitan area of Rome and were instructed to fast overnight for at least 12 h before their scan and to abstain from caffeine, nicotine or alcohol intake. From the time of entry in the hospital, the patients were in an air-conditioned environment at about 22°C. After the injection of ^18^F-FDG the patients rested quietly in a room at 24°C for 60 minutes until PET/CT measurement. Subjects were neither warmed nor instructed to avoid cold before the PET/CT examinations. Data on sex, age, weight, height, body mass index (BMI), plasma glucose level and cancer status (non active = negative finding on PET/CT scan; active = PET/CT scan abnormal uptake related to malignancy) were obtained for all patients. If the diagnosis was not available, it was classified as undetermined.

The patients with BAT activity constituted the study group. At least two study-date, sex adjusted matched subjects without documented BAT, were included as controls. Patients were considered to have ^18^F-FDG BAT when the following criteria were met: 1) ^18^F-FDG uptake was in the paravetebral/cervical, supraclavicular, mediastinal, paravertebral/dorsal, and abdominal or perirenal areas, unrelated to muscle, joints and pathological findings; 2) ^18^F-FDG uptake had a maximum standardized uptake value (SUV) of 2.0 g/ml or greater (an indicator of ^18^F-FDG uptake intensity); and 3) the tissue corresponded to the density of adipose tissue on CT (–250 to –50 Hounsfield units). For patients exhibiting ^18^F-FDG BAT, the mean and maximum SUVs for each identified depot were determined using a commercial fusion software (MIM software; MIMvista Corp., Cleveland, OH). The volume of ^18^F-FDG BAT was quantified by autocontouring each identified individual BAT depot. BAT glucose-uptake activity was calculated by multiplying the BAT volume by the mean SUV. Positive ^18^F-FDG BAT uptake patients, who underwent serial examinations during the study period with at least one negative scan were identified, and environmental variations occurring at the first negative scan were recorded. Outdoor temperature and day length in Rome for the day of the scan and up to three days before were obtained from the Ministero della Difesa, Italia, Servizio Meteorologico (http://www.meteoam.it/).

### Statistics

All analyses were performed using the SPSS software version 18. To assess the differences between patients with and without BAT ^18^F-FDG uptake, Student’s *t* test was used for normally distributed variables, the Wilcoxon-Mann Whitney *U* test was used for non-normally distributed variables, and the X^2^ test was used for proportions. Relationships between variables were assessed by Pearson or Spearman analysis, as appropriate. Univariate and multivariate logistic regression analyses were used to test the significance of the associations between BAT and biological and environmental factors. Only those variables significant at univariate analysis were tested in multivariate analysis. All p values presented are two sided and those less than 0.05 are considered statistically significant.

## Results

**Figure 1:**
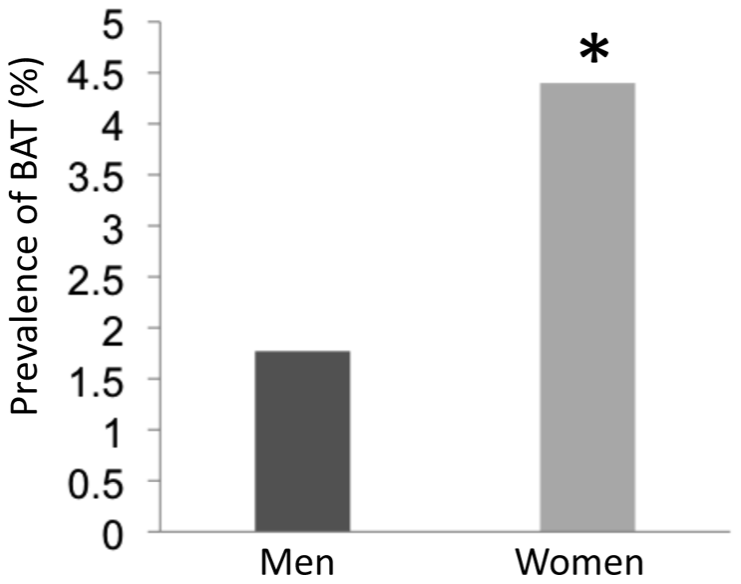
Prevalence of detectable BAT in men and women. The bars show the percentage of ^18^F-FDG males and females positive BAT in a total population of 6454 patients (3293 women and 3161 men). ★, p<0.001.

**Figure 2:**
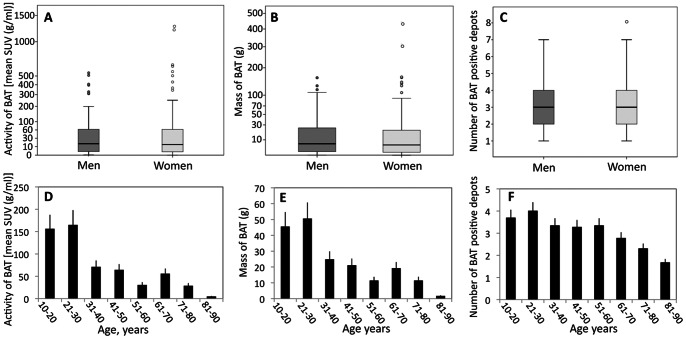
Mass, activity and number of BAT positive depots in men and women and effect of age on the occurrence of BAT. Panel A shows the mass of BAT in grams. Panel B shows the activity of BAT in grams times the mean SUV value in grams per milliliter. Panel C shows the number of BAT positive depots. The box plots indicate the lower quartile (lower line), median quartile (middle line), upper quartile (upper line), 1.5 times the interquartile range (lower and upper whiskers), and outliers (circles). Patients were divided into decades. The mean ± S.D. of activity (D), mass (E) and number of BAT positive depots (F) in each decade is shown.

We found ^18^F-FDG BAT in 217 of the 6454 patients (3.36%), the largest cohort of patients studied for BAT ^18^F-FDG PET/CT scans detection reported to date. Some of them underwent more than one ^18^F-FDG PET/CT scan and the positive scans were 278 among 8004 (3.47%). According to previous studies, the prevalence of positive ^18^F-FDG PET/CT scans was lower in men (1.77%; 56 of 3161) compared with women (4.88%; 161 of 3293), with a female to male ratio of 2.8∶1 (Figure 1). Age, anthropometrics, and metabolic characteristics of the patients with at least one positive scan compared with day matched BAT negative controls are shown in Table 1. The age and sex distribution of patients without detectable BAT was the same as that of the total population of patients undergoing PET/CT (data not shown). The BAT positive patients were most frequently women, younger, thinner and with lower plasma glucose levels compared with BAT negative patients. Among the BAT positive patients, the male subjects were significantly younger compared with the females (39.91±18.73 vs 47.39±15.77 years, p<0.009) and, although not statistically significant, had an higher BMI (24.16±3.57 vs 23.30±3.8 kg/m^2^) and fasting plasma glucose (96.79±16.12 vs 94.8±13.03 mg/dl). Both univariate and multivariate analyses showed that age, BMI and fasting glucose have an inverse correlation with ^18^F-FDG BAT evidence (table 2). The three predictors remained independently associated with BAT in women. In men, only age remained a significant independent determinant of BAT. The median amount of detectable BAT in the defined region of interest was 24.01±38.52 g (range, 0.054 to 159.57 g) in men and 24.69±53.16 g (range, 0.027 to 453.54 g) in women (Figure 2A). The median activity of BAT (Figure 2B) and the number of positive sites of active BAT (Figure 2C) were similar in both sexes but were skewed to higher levels in women than in men. The age range of the BAT-positive study group was 13–85 years and there was a trend for BAT activity (Figure 2D), mass (Figure 2E) and number of individual depots (Figure 2F) to decrease with increasing age. An evaluation of the anatomical distribution of BAT revealed that the most common location was the supraclavicular (87.55% of subjects), paravertebral/cervical (58.98%), paravertebral/dorsal (51.15%), followed by the mediastinal (35.48%) and abdominal or perirenal (5.52%).

**Table 1:** Clinical and physical characteristics of BAT-positive and date matched BAT-negative control patients.

	total	BATpositive	BAT negative	P value
n.	645	217	428	
Sex (female/male)	477/168	161/56	316/112	
Age (years)	56.25±15.96	45.4±16.8	61.7±12.3	<0.001
Weight (kg)	68.44±13.71	64.7±11.9	70.3±14.5	<0.001
Height (cm)	164.69±8.62	165.7±9.0	164.1±8.3	0.35
BMI (kg/m^2^)	25.23±4.73	23.5±3.7	26.0±4.9	<0.001
Glucose (mg/dl)	102.24±21.75	95.3±13.9	105.7±24.0	<0.001

Data are expressed as mean ± standard deviation (SD). Statistical differences between groups were analyzed by Student’s t-test.

**Table 2:** Univariate and multivariate analyses of factors associated with positive ^18^F-FDG BAT.

	Univariate analysis			Multivariate analysis								
	All patients			All patients			Males			Females		
Variable	Coefficient	Odds ratio(95% CI)	p value	Coefficient	Odds ratio(95% CI)	p value	Coefficient	Odds ratio(95% CI)	p value	Coefficient	Odds ratio(95% CI)	p value
**Age**(years)	−0.074	0.929(0.917–0.942)	0.0001	−0.065	0.937(0.924–0.950)	0.0001	−0.084	0.920(0.895–0.945)	0.0001	−0.059	0.942(0.927–0.958)	0.0001
**BMI**(kg/m^2^)	−0.145	0.865(0.827–0.905)	0.0001	−0.097	0.907(0.864–0.954)	0.0001	−0.108	0.898(0.793–1.017)	0.090	−0.90	0.914(0.865–0.965)	0.001
**Glucose**(mg/dl)	−0.035	0.966(0.954–0.977)	0.0001	−0.013	0.987(0.976–0.999)	0.033	0.006	1.006(0.990–1.022)	0.454	−0.021	0.979(0.965–0.994)	0.005

**Figure 3:**
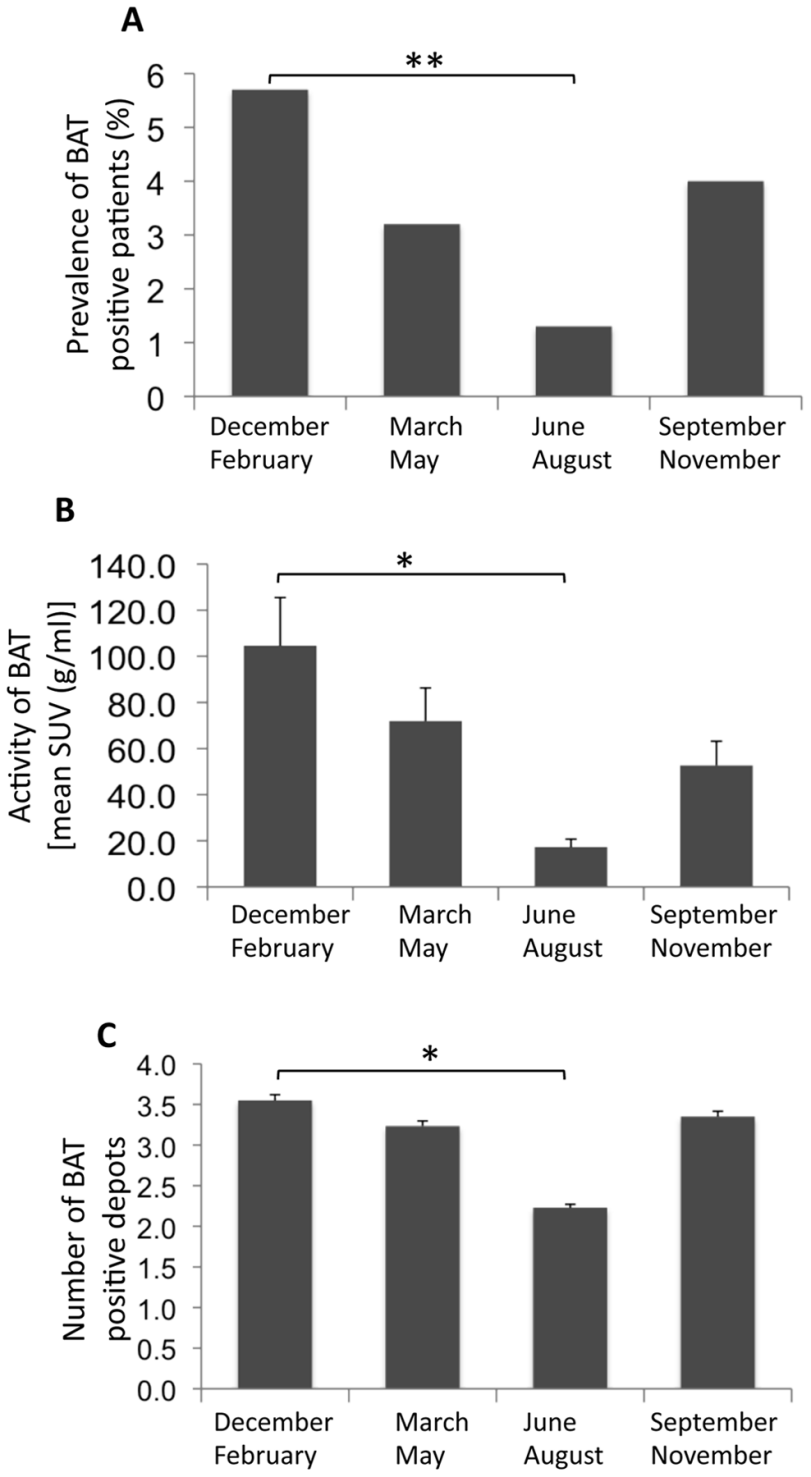
Seasonal variation in the occurrence of BAT. Panel A shows the percentage of BAT positive patients referred to the total population scanned in the indicated period. Panel B shows the activity of BAT in grams times the mean SUV value in grams per milliliter of the positive patients referred to the period indicated. Panel C shows the number of BAT positive depots referred to the period indicated. Significant effect of period assessed by either Wilcoxon-Mann-Whitney *U* test (★ p<0.01) or X^2^ test (★★ p<0.001).

The long-term effect of outdoor temperature on BAT expression was estimated by reviewing the dates of PET/CT scans in patients with detectable BAT as compared with the dates of the scans in all patients. The prevalence of patients with ^18^F-FDG positive PET/CT was highest in December-February, lower in March-May and September-November, and lowest in June-August (Figure 3A). The values of BAT mean activity (Figure 3B) and number of BAT positive depots (Figure 3C) paralleled the pattern of the proportion of patients with BAT activity.

**Figure 4:**
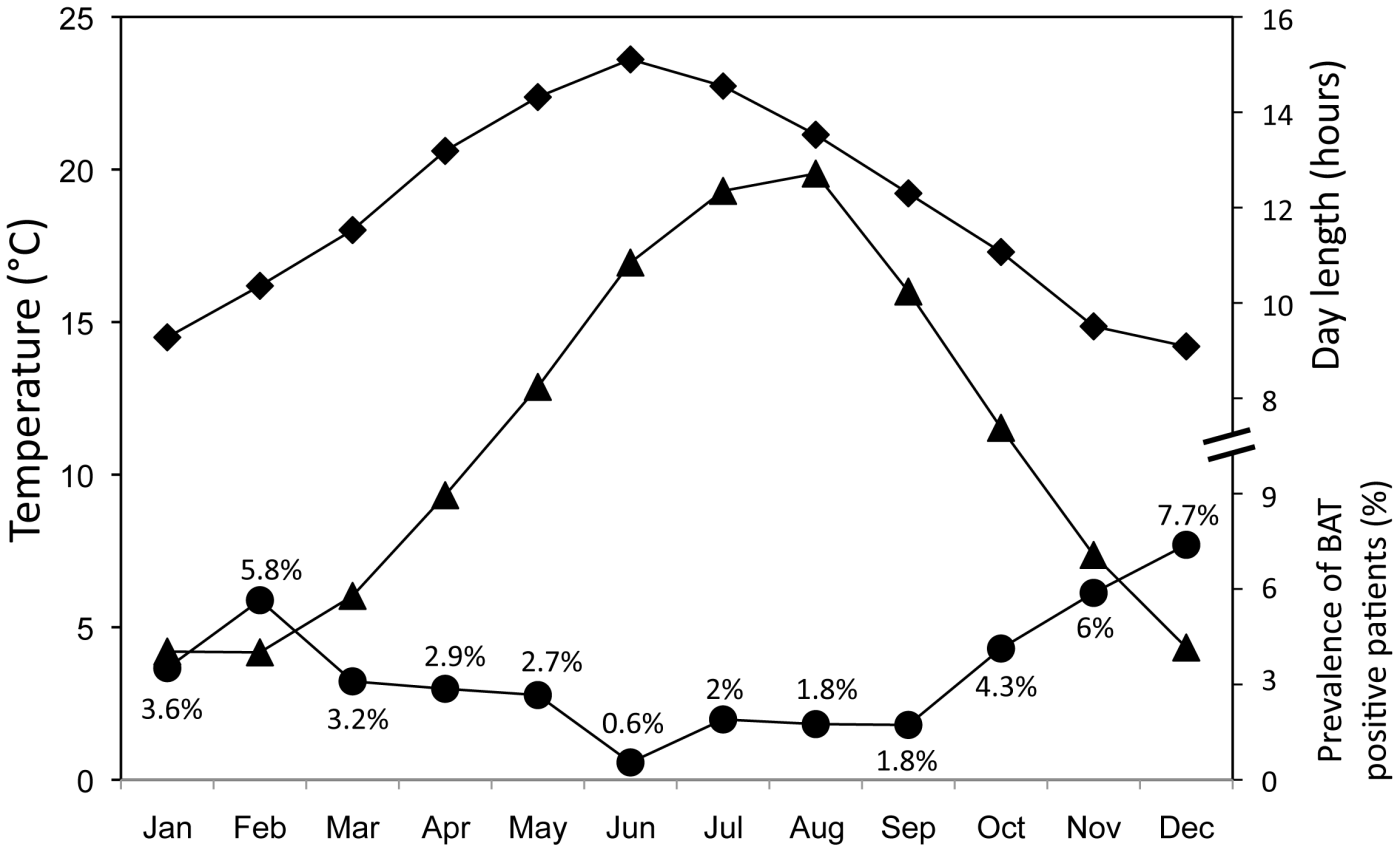
Average monthly mean values of ^18^F-FDG BAT. Prevalence (•) for the period January 2007-June 2010 and corresponding average minimum outdoor temperature (▴) and day length (♦).

**Figure 5:**
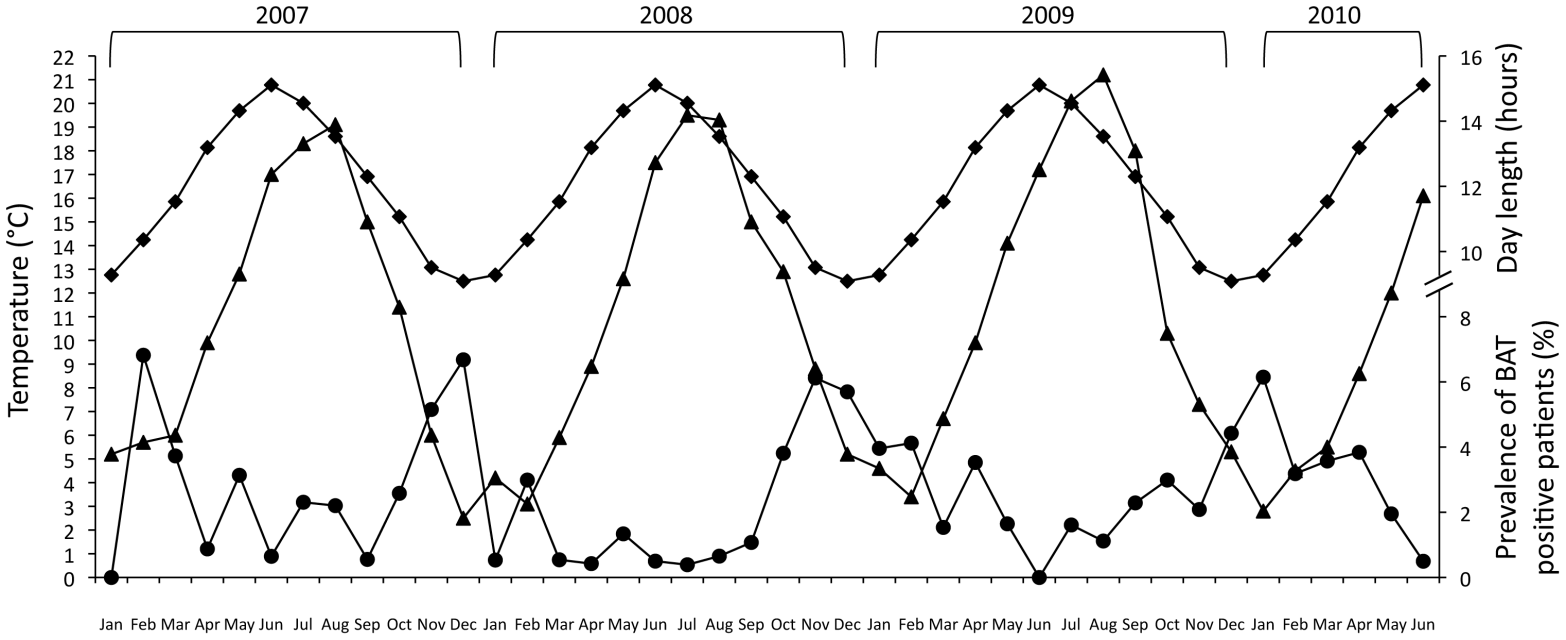
Monthly ^18^F-FDG BAT. Prevalence (•) of the monthly ^18^F-FDG BAT for the period January 2007–June 2010 and corresponding minimum outdoor temperature (▴) and day length (♦).

Figure 4 shows the average monthly incidence of BAT expression and its temporal relationship with the average monthly minimum temperature and day length as observed during the study period. The highest probability of observing^ 18^F-FDG BAT was in December, whereas the lowest probability was in June. The difference in the monthly number of patients in which BAT activity was detected correlated negatively with day length (r = −0.596; p<0.01; Spearman correlation) and ambient temperature (r = −0.527; p<0.01; Spearman correlation). In figure 5, we show the detailed monthly 3.5 year overall seasonal patterns of the prevalence of ^18^F-FDG BAT positive patients. There was a noticeable year-to-year overlapping in monthly ^18^F-FDG BAT pattern of expression. The variation (Δ) of the monthly prevalence of BAT, was inversely proportional with Δ of both monthly day length (p = 0.031) and minimum temperature (p = 0.032) with the variables set as dichotomous (−1 = decrease, +1 = increase, relative to the previous month). This trend was confirmed throughout the entire observation period. Among the 217 patients with ^18^F-FDG positive BAT, 99 (30 males and 69 females) had multiple scans during the survey period. We selected the first negative scan subsequent to the first positive scan. Outdoor temperatures either mean, minimal and maximum measured the day of the scan and 24 hours and 48 hours before, were significantly lower on the occasion when BAT was detected, and day length was shorter (table 3). There were no significant differences in fasting glucose levels, age, BMI or neoplastic status on the days when BAT was detected compared with the data collected on the days of negative scans. Univariate logistic analysis showed that day length and minimum outdoor temperature (the best predictive value at stepwise analysis) the day of PET-CT scan and 24 and 48 hours before were predictive of ^18^F-FDG BAT uptake (table 4). At multivariate logistic analysis, only minimum outdoor temperature 48 hours before the day of the scan remained significant.

**Table 3:** Characteristics of 99 patients showing positive ^18^F-FDG BAT uptake at first scan and their characteristics at first subsequent negative scan; day length and temperatures the day of the scan and 24 h and 48 h before.

Variable	BAT positive	BAT negative	p value
Age (years)	44.49±17.25	44.6±17.33	N.S.
BMI (kg/m^2^)	23.56±3.9	23.77±3.9	N.S.
Plasma glucose (mg/dl)	95.13±13.9	92.31±17.63	N.S.
Neoplastic status (active:non active)	53∶46	58∶41	N.S.
Day length (hours)	11.26±2.03	12.61±2.02	<0.001
Average temp (°C)	12.64±6.4	16.92±6.7	<0.001
Min temp (°C)	8.18±6.0	12.08±6.15	<0.001
Max temp (°C)	17.51±7.09	22.16±7.7	<0.001
Average temp (°C) 24 h before the day of the scan	12.48±6.54	17.00±6.73	<0.001
Min temp (°C) 24 h before the day of the scan	7.93±5.96	11.94±6.04	<0.001
Max temp (°C) 24 h before the day of the scan	17.32±7.34	22.51±7.4	<0.001
Average temp (°C) 48 h before the day of the scan	12.43±6.33	17.08±6.38	<0.001
Min temp (°C) 48 h before the day of the scan	7.73±5.75	12.06±5.84	<0.001
Max temp (°C) 48 h before the day of the scan	17.53±7.18	22.66±7.33	<0.001

Data are expressed as mean ± standard deviation (SD). N.S., not significant. Statistical differences between groups were analyzed by Student’s t-test.

**Table 4:** Analysis of putative predictors of detectable BAT based on PET-CT scanning of 99 patients with positive ^18^F-FDG BAT uptake at first scan compared with the first subsequent negative scan.

	Univariate analysis			Multivariate analysis		
Variable	Coefficient	Odds ratio(95% CI)	p value	Coefficient	Odds ratio(95% CI)	p value
Day length (hours)	−0.315	0.730(0.633–0.842)	0.0001	−0.170	0.844(0.684–1.047)	0.113
Min temp (°C) the day of the scan	−0.103	0.902(0.859–0.947)	0.0001	0.002	1.002(0.884–1.135)	0.98
Min temp (°C) 24 h before theday of the scan	−0.109	0.896(0.853–0.942)	0.0001	0.064	1.066(0.894–1.271)	0.478
Min temp (°C) 48 h before theday of the scan	−0.127	0.881(0.836–0.929)	0.0001	−0.149	0.862(0.74–0.993)	0.040
Neoplastic status (active:non active)	0.250	1.284(0.729–2.263)	0.387	–	–	–

## Discussion

The increasing prevalence of obesity in developed countries has become a difficult task. The strategies and methods used to retard obesity in recent decades have failed. The discovery that substantial amounts of functional BAT are retained in adult humans [3]–[8] provides a potential means to limit the obesity problem. BAT is a metabolic active organ responsible for non-shivering thermogenesis in mammals. BAT can convert extra energy preserved in fat to heat by means of uncoupling oxidative phosphorylation in mitochondria [2]. This property is particularly important for humans as extra energy intake is a major factor contributing to development of obesity. An important feature of BAT is that its activation is readily manipulated by physiological, environmental and pharmacological interventions. As a result, BAT might be an ideal target for novel anti-obesity drug development and behaviour/educational therapies. A greater understanding of BAT function could thus help to develop treatment strategies for obesity. Here we report the characteristics of BAT expression in a large cohort of adults undergoing PET/CT, specifically focusing on the impact of photoperiod and temperature, key factors for the determination of BAT function.

We found a 3.47% prevalence of ^18^F-FDG BAT. This value is halfway the prevalence reported in other studies involving large cohorts of patients, that ranges from 1.72% to 6.8% [16]. Although the exact characteristics of the study populations of the previous studies in terms of age, male to female ratio, BMI, fasting plasma glucose and exposure to drugs are not known, a possible explanation for these differences might reside in the outdoor temperature and day length. Environmental temperature is of great importance for the outcome of a ^18^F-FDG-PET study. Interestingly, the average temperatures of the metropolitan area of Rome during all time of year were higher than the temperatures recorded in the latitudes where the other large cohort studies, lasting at least one year and reporting higher prevalence of BAT, were conducted [16]. Accordingly, when the average outdoor temperature was higher compared with Rome, the prevalence of BAT was lower [13], [16] and, on the contrary, when the average outdoor temperature was lower compared to Rome, the prevalence of BAT was higher [4], [9]–[11], [14], [15]. We confirmed that women have a greater prevalence of BAT [4], [9], [10], [13], [14], [17], [18], although activity, mass and number of BAT positive depots did not differ among sexes. BAT expression was negatively associated with age, BMI and fasting plasma glucose levels. This confirms that, in general, BAT expression declines with aging, adiposity and glucose. Interestingly, we found that the association of BAT expression with BMI and glycemia was lost in men and conserved in women while the predictive value of age persisted in both sexes. These differences may be caused by differences in study groups. In our cohort, men showing BAT were significantly younger than women and age is one of the strongest single predictor of BAT expression. Conversely, BAT positive male subjects of our study group had higher BMI and fasting plasma glucose values compared with females. It could be argued that age would have progressively greater influence on BAT expression when other predictors, like BMI and plasma glucose, reduce their predictive power. Sexual dimorphism in endocrine control of BAT, possibly related to sex hormones, may also contribute to explain the gender differences in the strength of the determinants of BAT expression [2], [19]. One striking finding of this study is the seasonal variation in BAT expression in terms of prevalence, number of depots and activity. In agreement with a previous report [10], we raise the question whether it is the prevailing ambient temperature or photoperiod that may be the primary factor determining BAT activity; in our study, the seasonal variation of BAT expression was closely related to both photoperiod and temperature. While there is a great amount of data on the influence of temperature on BAT expression, somewhat intriguing is the finding of the recurrent inverse relationship of the variation of day length with the prevalence of BAT both in terms of correlation and predictive value observed during this long term retrospective study.

A majority of mammals shows marked seasonal variations in body weight, including BAT weight, which appear to be driven by changes in photoperiod in addition to and even irrespective of temperature [20], [21]. The exact mechanisms as to how photoperiod regulates the recruitment of BAT are not available currently. However, there are several clues that suggest the mechanisms involved. Short photoperiod enhances sympathetic tone [22], [23], [24] that, through β-adrenergic pathway, is the most important and the most well studied stimulation of BAT activity [2]. Prolactin, a hormone highly responsive to changes in day length [25], [26] that controls brown adipocyte differentiation and growth [27] through prolactin receptors [28], [29], may have a role in regulating BAT development and thermogenic activity [30]. The physiology of BAT is readily regulated by melatonin, the molecule that transduces the signal of the environmental photoperiod to synchronize endogenous functions with daily and seasonal rhythms [31]. Melatonin, not only increases recruitment of brown adipocytes but also elevates their metabolic activity in mammals. It is speculated that the hypertrophic effect and functional activation of BAT induced by melatonin in small mammals, may likely apply to humans. Short photoperiods characteristically predict upcoming cold weather and food scarcity; this information is essential for hibernators as it is a signal to recruit BAT. Further studies correlating catecholamines, prolactin, melatonin and other hormones potentially involved in the BAT expression and photoperiod are warranted.

Furthermore, in light of the recent finding of the abundance of BAT activity in the epicardial adipose tissue [32], [33], a visceral fat depot of the heart [34], [35] with proposed role both in the protection of heart from hypothermia [32] and in the pathogenesis of coronary artery disease [36], a variation of BAT in the epicardial fat which parallels the variation of BAT in the classical locations of BAT expression could contribute to the well recognized seasonal variation in the incidence of heart diseases.

We did not find a significant association between active neoplastic status and BAT at repeated ^18^F-FDG PET/CT scan. However, in patients living in tropical areas where the consistently warm outdoor temperature and the limited day length variation reduce the effect of cold environment and length of day on BAT, neoplastic status, more than temperature and day length, is a critical determinant of BAT activity [37].

In conclusion, our retrospective study describes the seasonal variation in BAT activity in a large cohort of patients coming from the temperate climate zone of Rome. We confirm and extend previous findings indicating the strong influence of both photoperiod and ambient temperature on BAT expression and function. The current obesity epidemic reflects multiple interactions between our lifestyle and inherited genetic background. Clearly, the type of diet consumed and the lack of physical activity will promote obesity. Daily long photoperiod exposure, alterations of ambient temperature, also because of the fact that the majority of humans dwell in thermoneutral conditions, are currently experienced by most humans. All these conditions may contribute to the constantly increasing prevalence of obesity in developed countries because of the negative effects on BAT expression. Our results highlight new approaches to reduce obesity by means of targeting BAT through both pharmacological interventions and environmental modifications.

We acknowledge several limitations to this study. First, the study evaluated detectable, active BAT under routine clinical imaging conditions, and so the incidence reported is not directly comparable to the findings in which adult volunteers had been intentionally cooled to determine maximum detectability of BAT by PET [5], [8]. Second, although consistent with previous reports [4], in our series there was no association of BAT uptake with oncologic diagnoses, all scans were performed for cancer surveillance, raising the possibility of physiological differences between some cancer patients and normal subjects.
